# La-CTP: Loop-Aware Routing for Energy-Harvesting Wireless Sensor Networks

**DOI:** 10.3390/s18020434

**Published:** 2018-02-02

**Authors:** Guodong Sun, Xinna Shang, Yan Zuo

**Affiliations:** 1 School of Information Science and Technology, Beijing Forestry University, Beijing 100083, China; shangxinna@buu.edu.cn; 2 Embedded Networked Systems Lab, Beijing Forestry University, Beijing 100083, China; 3 College of Robotics, Beijing Union University, Beijing 100101, China; 4 Department of Computer Science and Technology, Dalian Neusoft University of Information, Dalian 116023, China; zuoyan@neusoft.edu.cn

**Keywords:** energy-harvesting wireless sensor networks, loop-aware routing, parent updating, adaptive beaconing

## Abstract

In emerging energy-harvesting wireless sensor networks (EH-WSN), the sensor nodes can harvest environmental energy to drive their operation, releasing the user’s burden in terms of frequent battery replacement, and even enabling perpetual sensing systems. In EH-WSN applications, usually, the node in energy-harvesting or recharging state has to stop working until it completes the energy replenishment. However, such temporary departures of recharging nodes severely impact the packet routing, and one immediate result is the routing loop problem. Controlling loops in connectivity-intermittent EH-WSN in an efficient way is a big challenge in practice, and so far, users still lack of effective and practicable routing protocols with loop handling. Based on the Collection Tree Protocol (CTP) widely used in traditional wireless sensor networks, this paper proposes a loop-aware routing protocol for real-world EH-WSNs, called La-CTP, which involves a new parent updating metric and a proactive, adaptive beaconing scheme to effectively suppress the occurrence of loops and unlock unavoidable loops, respectively. We constructed a 100-node testbed to evaluate La-CTP, and the experimental results showed its efficacy and efficiency.

## 1. Introduction

Energy resource is a dominant constraint in wireless sensor networks (WSNs), so prolonging the system lifetime is always an important topic for real-world applications [1,2]. Recently researchers have attempted to employ the environmental (ambient) energy to drive the wireless sensor nodes. A variety of environmental energy sources exist in nature, such as solar energy, wind energy, RF energy, vibration energy, and hydrokinetic energy [3,4,5,6,7]; some of them are renewable and can be easily captured and stored in rechargeable battery. By harvesting such renewable environmental energy to power wireless sensor nodes, the users do not need to frequently replace (or manually charge) the battery modules of their nodes, and then they could achieve longer or even perpetual system operation [8,9,10,11]. In a WSN, if all the nodes or a part of them are driven by environmental energy, it is usually called an energy-harvesting wireless sensor network (EH-WSN, in short). EH-WSNs have recently attracted increasing attention from academia and industry.

For the energy-harvesting node in EH-WSNs, its energy consumption rate is far higher than its energy charging rate. An immediate, unavoidable consequence is that the energy-harvesting node cannot afford to keep active all the time, because it must stop working—leaving the network—to replenish its energy. The absence of recharging nodes, even temporary sometimes, is very likely to result in a segmented network topology, which could be recovered only until some of those recharging nodes return to the network with sufficient energy capacity. In such an intermittently-connected network, a node will lose its current forwarder (also called next-hop or parent node), if the recharging process comes on at that forwarder. If the parent-losing node cannot reestablish valid routing paths, its packet will have to be dropped after many failed retries, wasting the scarce resources of energy and bandwidth. Though unreliable, low-power wireless channels and energy-harvesting processes are both likely to break off forwarding paths in EH-WSNs, the latter will impact the network performance and efficiency more severely than the former does. Therefore, recovering or re-determining valid forwarding path is an urgent and challenging mission for EH-WSN applications due to their intermittent and random nature in connectivity.

**Figure 1:**
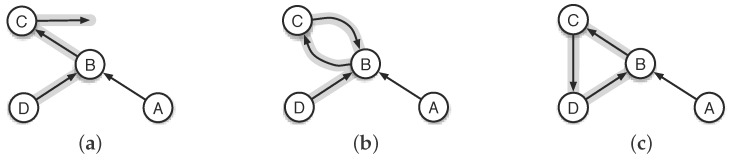
Illustration of the typical routing loops. (**a**) no loop; (**b**) 2-node loop; (**c**) 3-node loop.

In the designs of practical EH-WSN routing protocols, the control of routing loops is a crux of establishing and maintaining throughput-high and cost-aware forwarding paths. Loops are most likely to occur during the routing path re-establishment of various networks, whether the routing protocols in use are designed based on the link state, the distance vector, or the application-specific gradient information [12,13,14,15,16,17]. A loop will come into network if a node chooses one of its upstreaming nodes as forwarder. Figure 1a shows a loop-free network. For the two cases shown in Figure 1b,c, however, the packets generated by nodes *A* and *D* will be locked in the network, never arriving at the destination, because node *C* mistakenly chooses *B* and *D* as its forwarders in the two cases, respectively. Basically, the unwise forwarder selection is due to the local network status changes that cannot be timely shared within the neighborhood after they happen. The 2-node loop shown in Figure 1b is easy to be identified and corrected by forcing each node not to send packets to its previous-hop node. However, the *n*-node (n≥3) loops are hard to be shunned or removed, unless each node can exactly know all its upstreaming nodes in a way of taking no account of the demand for more network resources and the risk of performance deterioration.

In contrast to traditional duty-cycled WSN [18,19], EH-WSNs are more prone to experiencing long-term loops, because the recharging periods of energy-harvesting nodes are usually heterogeneous and random, and because the unreliability of low-power wireless links will also compound the bad situation. Additionally, in the testbed experiments of EH-WSN, we found that the local network status varies frequently and the poor network connectivity caused by such variations could spread over the whole network quickly. Therefore, the routing protocols for duty-cycled WSNs have little effect on EH-WSNs in terms of loop handling (Section 2 will detail the reasons behind). We also noticed that existing routings for EH-WSNs are based on rigid models or assumptions and pay much theoretical attention on promoting the energy efficiency in forwarder selection. In particular, they did not comprehensively touch on the issue of great practical significance [20]: plenty of network resources might go to waste once loops occur in EH-WSNs, yet the recovery of valid forwarding paths cannot be necessarily accomplished in short term.

In this paper, we will present a loop-aware routing protocol (La-CTP) for practical EH-WSN applications. The experimental results based on a 100-node testbed showed that La-CTP can deal with loops effectively with low cost and yield desirable throughput. To the best of our knowledge, our work is the first attempt to design practicable loop-aware routing protocol for EH-WSNs. The major contributions of our study include: (1) we design La-CTP based on CTP [21], which takes care of almost all the networking aspects of traditional WSNs, and then La-CTP has strong practicability in engineering; (2) we analyze the structure and the disadvantages of CTP in an anatomical way, providing protocol designers and engineers with clear understanding of the imperceptible operation mechanism of CTP; (3) the proposed La-CTP uses a new parent update metric to effectively restrain the occurrence of loops, and employs a proactive and adaptive beaconing scheme to unlock loops as soon as possible; and (4) we construct a 100-node testbed to simulate the operation of EH-WSN, and conduct extensive experiments with typical scenarios and deep analyses to verify the performance of La-CTP in handling loops.

The remainder of this paper is organized as follows. Section 2 introduces some existing works related to ours. Section 3 briefly introduces the architecture of CTP. Section 4 analyzes the problems and shortcomings of CTP in dealing with loops in EH-WSNs. Section 5 details the design of La-CTP. Section 6 evaluates La-CTP and compares it with CTP under different network deployments. Finally, Section 7 concludes this paper and charts some future directions of further improving La-CTP.

## 2. Related Work

There have been a great amount of routing protocols or algorithms [22,23,24] proposed for WSNs, but they cannot fit in well for practical EH-WSNs, especially being incapable of handling loops [25,26].

Similar to the energy harvesting scenarios, duty-cycled WSNs [18,27,28] allow nodes to switch their status from working to sleeping, and back to working. In the duty-cycled scenario, the node switches its status according to a working schedule or a duty cycle that is usually configured in system initialization. The working schedule consists of active slots and sleeping slots, and the span of a single slot is about tens of or hundreds of milliseconds. Dividing the operation time into slots allows the node to reduce the energy consumed by the idle listening of channels, while not impacting the transmission delay too much. There are three main differences between duty-cycled WSN and EH-WSN. First, compared to the duty-cycled nodes, the EH-WSN nodes could leave the network for energy replenishment for a longer time, say, hundreds of seconds or even much longer, which depends on the capacity of energy harvesting per se. Second, in duty-cycled scenario, the node can be waked up by the routing protocol to work, if it is required to participate into packet forwarding. For the EH-WSN scenario, however, a node cannot re-join the routing process in any way, until it completes battery recharging. Third, in practice, the recharging period of an EH-WSN node is likely, but far from certain, to be accurately estimated [29], because the environmental energy source in use is time-varying in output power and hard to be modeled in advance. In duty-cycled WSNs, the nodes often depend on global time synchronization such that all nodes operate strictly by their working schedules and each node can precisely know the working schedules of its neighbors.

In recent, researchers have studied or designed routing protocols and algorithms for emerging EH-WSNs. CHESS [30] (Communication using Hybrid Energy Storage System) is an early routing metric for EH-WSNs, which considers the efficiency of energy storage and usage and focuses on the management of energy recharging. In [31], an opportunistic routing protocol, called EHOR, was proposed. EHOR first divides the nodes into different groups and considers the residual energy in forwarder selection. EHOR was evaluated with a simulator. In [32] an opportunistic routing protocol (AOR) was presented for EH-WSNs. However, AOR depends heavily on the geographic information of the deployment, without considering the possible loops. Liu et al. [33] designed a scheme for fair and high throughput data collection in the case of involving the EH-WSN nodes. Hieu et al. [34] designed a stability-aware geographic routing for EH-WSNs; they aimded to improve the network throughput while considering the harvesting capacity and the residual energy. Wu et al. [35] proposed a gradient information-aware, hierarchical clustering algorithm. The authors believe that no loops will occur, as the forwarder selection always refers to the gradient information that should keep increasing along the delivery path to sink. However, their numerical experiments did not consider the effect of dynamic wireless channel and random recharging durations. Gul et al. [36] collected data from energy-harvesting nodes that were organized into a single-hop network and constructed the models for throughput optimization. Several other routing approaches (summarized in [37]) were proposed for EH-WSNs and they were dedicated to improving the energy efficiency by balancing the energy consumption.

Most of the existing works put more effort on the energy efficiency improvement and on the recharging regulation during packet routing. Furthermore, being evaluated by simulation experiments, they were usually designed as a pure routing layer, without the support from or the compatibility with other indispensable low-level communication layers. Therefore, they might not suit the real-world EH-WSNs easily. They also do not fully consider the effect of routing loops, which will waste a great amount of network resources in practice.

## 3. Overview of CTP

There have been a lot of WSN routing protocols proposed in literature in recent years; of them, CTP is one of the most important routing protocol widely considered in both real-world WSN deployments and academic studies. CTP is a tree-structured protocol which collects data from wireless sensing nodes in a many-to-one way, and it has been one part of the protocol stack of TinyOS (http://tinyos.stanford.edu/tinyos-wiki) (an operating system for tiny wireless embedded system). CTP aims at providing reliability, robustness, efficiency, and hardware independence in delivering sensory data. To see how CTP might work, we will next introduce its architecture and some major schemes involved.

**Figure 2:**
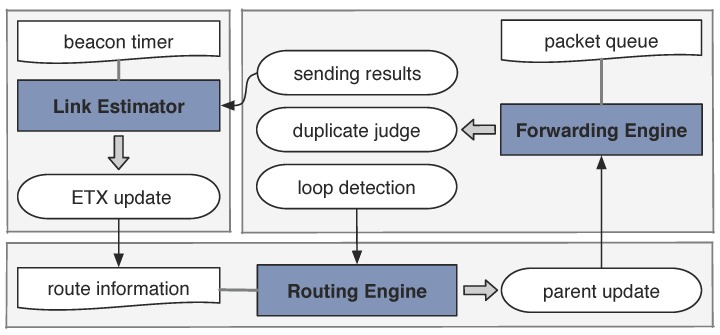
Brief description of the architecture of CTP.

CTP includes three collaborative components: the Link Estimator, the Forwarding Engine, and the Routing Engine. Figure 2 shows the architecture of CTP. The **Link Estimator** is responsible for estimating the single-hop ETX value [38] from the local node to its neighbor. ETX (expected transmission count) is a metric measuring the quality of two-way link. Such estimations are performed according to the beaconing scheme that controls a beacon timer. The **Routing Engine** uses the estimated ETX values and the network-level information (including the feedback from the Forwarding Engine), aimed at deciding which neighbor is more desirable to serve as the forwarder or the parent node. The **Forwarding Engine** maintains a packet queue and decides when and how to send the locally-generated or the passing-by packets. The critical function of the Forwarding Engine is to detect packet duplicates and routing loops.

In short, the three components of CTP form a closed-loop control of information flow: CTP dynamically updates its routing information according to the link and network states collected at runtime. The implementation of CTP involves three supportive techniques, which are critical for it to achieve the performance goals, and they are as follows.

1. *Agile Link Estimation*: to estimate the radio link quality, CTP uses the four-bit link estimator which profiles the dynamics of the physical, the data-link, and the routing layers. Especially, CTP updates such estimations as quickly as every five packet receptions in order to ensure the accuracy.
2. *Datapath Validation*: topology changes often lead to transient loops in network, which in turn cause significant packet drops, wasting the precious network resources. In detail, CTP uses ETX as the metric for measuring both the single link and the path to the sink. The ETX value of a path is the sum of the ETX values of all the links along this path. Before a node forwards a packet, it will examine whether the ETX value of the passing-by packet is smaller than its local ETX; if true, CTP thinks that routing loops have happened to the previous-hop node.
3. *Adaptive Beaconing*: by exchanging control beacons in a series of time intervals, CTP updates stale routing structure. The adaptive beaconing scheme, improved on the basis of Trickle [21], breaks through the traditional trade-off between the fast recovery of connection and the low cost, and it can control when to reset the timer interval of sending beacons, according to the routing cost gradient that is maintained locally by each node.

CTP is a well-engineered routing protocol for WSNs, synergistically integrating the 4-bit link estimator, the Trickle beaconing scheme, and the ETX-based routing gradient computation. In recent, CTP has developed into one of the most successful routing protocols for collecting data from academic and industrial wireless networks [39,40], and it has also become the de facto standard for data collection in WSN applications [41,42,43].

## 4. Dissection of CTP in EH-WSN

We conducted three artificially-controlled, small-scale simulation experiments, which are hereafter labelled with T-I, T-II, and T-III, respectively. By these three experiments, we try to (1) unravel the basic control principles of CTP; (2) investigate the reaction of CTP when the recharging nodes are going out of and back to the network; and (3) evaluate the performance of CTP and its sticking points in recovering from loops.

### 4.1. Experiment T-I

**Figure 3:**
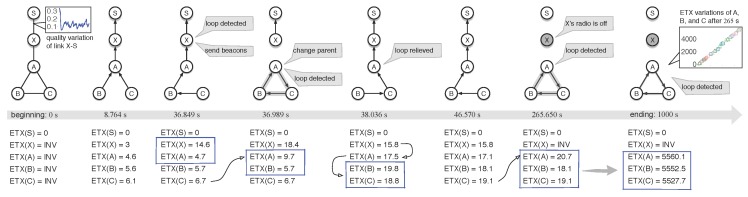
Snapshots of the CTP in experiment T-I, during which node *X* is shut down at the 260th second. The time point below each topology timestamps the current experiment snapshot. The ETX values listed are the route ETX (routing gradient) instead of the link ETX.

Experiment T-I involved five wireless nodes and ran for 1000 s. Figure 3 shows several chronological snapshots of T-I; the most left subfigure shows the initialized network topology where node *S* is the root (or the sink) of collecting the data generated by nodes *A*, *B*, *C*, and *X* every 10 s. Especially, we set a lossy and time-varying link between *X* and *S* (the link PRR is 30% on average), in order to examine the effect of link quality attenuation on its upstream data routing. When T-I went to the 265th second, the radio module of node *X* was turned off, i.e., it departed from the network. Next we will chronologically describe what happened in T-I.

[T-I: 8.764 s]: at this time, the routing tree structure has been established and CTP runs normally without any routing inconsistency—each child node is higher than its parent node in terms of ETX value, as can be seen from the ETX values in Figure 3 that are listed below the snapshot of T-I: 8.764 s.

[T-I: 36.849 s]: during the quality of link $l_{XS}$ deteriorates continuously from the beginning till this time point, the route ETX of node *X* increases from 3 to 14.6. When this time point is hit, *X* first receives a packet sent out by *A* and finds that its local route ETX value, ETX(*X*) is greater than its child’s route ETX value, ETX(*A*) = 4.7, indicating that routing inconsistency occurs. CTP is designed to determine the loop occurrence by comparing ETX values. In detail, when a packet arrives, the receiving node will immediately check the route ETX field of this packet; if the in-packet ETX is less than the local route ETX, the receiving node will conclude that there exists a loop or a routing inconsistency; and then it will immediately launch its Trickle-based adaptive beaconing scheme to notify its neighbors, in hope of repairing the broken forwarding path. Specifically, node *X* will broadcast its routing control beacons 64 ms after it detects routing inconsistency.

After node *A* receives the beacon from *X*, therefore, *A* decides to update its parent by looking up its routing table locally maintained. Now, the routing table of *A* has two potential parents, *B* and *C*. However, *A* will choose *C* as its new parent because *A* knows that *B* is its child. In addition, then, *A* updates its route ETX from 4.7 to the value of 9.7, which equals the sum of the route ETX of *C*, 6.7 and the link ETX of link $l_{CA}$, 3.0.

[T-I: 36.989 s]: just after finishing parent update, node *A* receives a packet from *B* and detects a loop event, because ETX(*A*) = 9.7 > ETX(*B*) = 5.7.

[T-I: 38.036 s]: before this moment, ETX(*X*) has changed from 18.4 down to 15.8. Once the decrease in local route ETX is beyond 2.0, the node must broadcast its latest routing status in its neighborhood, as needed by CTP. At this moment, node *A* has detected loops and decides to update its parent; and finally, *A* chooses *X* as its new parent and updates its local route ETX(*A*) with the value of 17.5. Very close behind the routing beacons of *A* triggered by the parent update event, nodes *B* and *C* also update their routes. From now till the time point of 265 s, the routing tree works smoothly—everything is under the control of CTP.

[T-I: 265.650 s]: we turn off the radio of *X* at time point 260 s; in other words, the departure of *X* from the current network makes *A* lose the next hop. Now, *A* has two potential parents to forward its data, nodes *B* and *C*. However, *A* will update its parent with *C*, instead of *B*, because *A* knows *B* is its child. Hence, *A* finally updates its route ETX with the value of 20.7, by referring to the current ETX(*C*). Obviously, loop ensues without any hesitation and can never be unlocked by CTP until T-I ends at time point 1000 s.

By comparing the local ETX and the ETX of the packet received, node *A* can detect the routing inconsistency the moment it receives a packet from *B*. Unfortunately, *B* has no choice as to parent update because it cannot choose its previous hop, *C*, as its next hop; therefore, *B* has to update its route ETX according to its parent, *A*. Similarly, *C* also has to update its route ETX because its parent is *B*. The three nodes, *A*, *B*, and *C* will always stay in the loop, even though they are able to detect loops at runtime. CTP has no any back-pressure schemes to notify upstream nodes about the loop status. Noticeably, once loops are identified, the nodes will always increase their own route ETX to satisfy the child-parent gradient rule. From the most right subfigure of Figure 3, we see that the ETX values of these three nodes keep going up after node *X* is removed out, and that all of them move beyond five thousand at the end of experiment T-I.

**Remark** **1.** CTP can work smoothly—removing loops— as long as the network always keeps connected. But it is nearly incapable of preventing the already-happening loops caused by the departure of recharging nodes from developing.

### 4.2. Experiment T-II

**Figure 4:**
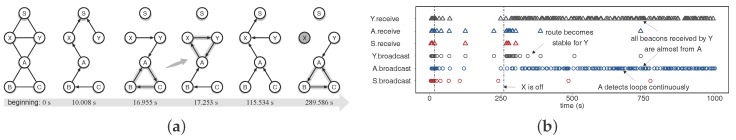
Snapshots and beaconing of the CTP in experiment T-II, during which node *X* is shut down at the 260th second. The qualities of link $l_{XS}$ and $l_{YS}$ in T-II are the same as that of link $l_{XS}$ in T-I in terms of dynamics. In the beaconing plots, for the clarity, we only show randomly-sampled 20% of the points after time 260 s for two kinds of beaconing events, “A.broadcast” and “Y.receive”. (**a**) critical snapshots; (**b**) route beaconing.

Experiment T-II involved six nodes; its initialized topology and some critical chronological snapshots are shown in Figure 4a. In T-II, links $l_{XS}$ and $l_{YS}$ were both set to be unreliable with the link dynamics similar to link $l_{XS}$ in experiment T-I. We had expected that node *A* could find a new route to *S* through *Y*, even when node *X* would be turned down at time point 260 s.

[T-II: 16.955 s]: the first loop happens due to the invalid links $l_{XS}$ and $l_{AX}$. The route ETX values of nodes *A*, *X*, and *Y* are all increasing, which further leads to the continuous increases in ETX at nodes *B* and *C*.

[T-II: 17.253 s]: the first loop containing nodes *A*, *B*, and *C* is successfully released just 258 ms after its happening, which is thanks to link $l_{AX}$ which turns into validity during that term. Nevertheless, another loop is formed with nodes *A*, *X*, and *Y*, because both *X* and *Y* still do not have available links connecting with *S*. The behavior of these two successive loops is like a raging tornado which moves from place to place.

[T-II: 115.534 s]: the second loop is removed at this moment due to the recovery of link $l_{XS}$.

[T-II: 260 s]: node *X* is turned off, resulting in the loss of parent at nodes *A* and *Y*. Node *A* will possibly choose *B* as its parent once more and *Y* will choose *A* as its parent with no alternative: a new loop passing through *A*, *B*, and *C* is coming soon.

[T-II: 289.586 s]: until now, node *Y* has not made successful handshakes with *S* since *X* went away. Thus, *Y* finally chooses *A* as its new parent, without minding the loop that is happening to *A* at this very moment.

Figure 4b plots the beacons broadcasted and received by nodes *S*, *A*, and *Y* during T-II. The distribution pattern of these beacons offers extra details as to the loops shown in Figure 4a. We can see in Figure 4b that at the very beginning of T-II, all the three nodes broadcast and receive beacons frequently, aimed at establishing valid forwarding paths as soon as possible. After the routing inconsistency is removed after T-II: 17.253 s, each node thinks there has been a stable route for itself and then increases the interval of its beacon timer. That is why less beaconing events can been seen between the time points T-II: 17.253 s and T-II: 260 s.

Shortly after node *X* leaves the network at time point T-II: 260 s, *A* and *Y* both lose their next-hop and then launch the beacon broadcasting almost immediately, struggling to find out new available parent. Finally, *A* chooses *B* as its parent at time point T-II: 289.586 s, which gives rise to a loop that will never leave the network until T-II ends. Unfortunately, this loop attracts the route seeker *Y*, making *Y* unhesitatingly become a child of *A*, even though *A* continuously broadcasts beacons of speaking out its loop detection. Node *Y* persists in its choice, thinking that it has already found a steady route provided by *A*. Consequently, *Y* does not reset (shorten) its beacon timer for parent update, which can be figured out, in Figure 4b, from the very small amount of the “Y.broadcast” events after time point T-II: 289.586 s. Another reason that *Y* abandons *S* as its parent comes from the behavior of *S*. When *S* does not receive any packets, it will assume no traffics and then certainly no loops in the network. With such an assumption made by CTP, *S* considers it unnecessary to frequently broadcast beacons into its neighborhood, and then passively continue to prolong its beacon timer exponentially; Figure 4b shows that the “S.broadcast” events are very sparse after time point T-II: 260 s so that *Y* does not have ample opportunities to timely learn about the routing status of *S*, especially when link $l_{YS}$ is time-varying.

**Remark** **2.** Only relieving one local loop is not necessarily helpful to the entire CTP network. The loop in EH-WSN acts like a tornado, not only moving around but also possibly attracting the “unstably-installed” surroundings—the close-by nodes without stable routes. Without knowing each other’s real situation, however, both the node attracted by loop and the passive potential forwarding node continually shorten their beacon timers and then create a sham normality of network operation, which in turn significantly reduces the chances of relieving loops.

### 4.3. Experiment T-III

**Figure 5:**
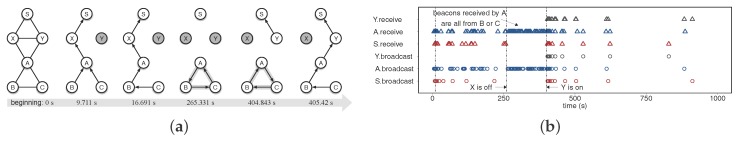
Snapshots and beaconing of the CTP’s operation in experiment T-III, during which node *X* is shut down at the 260th second and node *Y* is turned on at the 400th second. The dynamics of link $l_{YS}$ in T-III is the same as that of link $l_{YS}$ in T-II. In the beaconing plot, for the clarity, we only show randomly-sampled 20% of the points between the 260th and the 400th seconds for two kinds of beaconing events, “A.broadcast” and “A.receive”. (**a**) critical snapshots; (**b**) route beaconing.

Experiments T-I and T-II examined the case in which a node entered the recharging status and then left the network. Furthermore, we conducted experiment T-III which involved a node that went back to the network after completing the energy replenishment. Figure 5 shows partial results of T-III.

Before the time point of T-III: 260 s, as shown in Figure 5a, the network keeps connected and runs normally, even though node *C* changes its parent from *A* to *B* when the experiment goes to T-III: 16.691 s. After node *X* is shut down at T-III: 260 s, a loop is formed, going through *A*, *B*, and *C*, and has always been kept there until node *Y* appears in the network. By the analyses of experiment T-II, we have understood why turning off *X* can cause a loop through *A*, *B*, and *C*. After *Y* finishes its energy recharging, it goes back to the network and immediately starts probing, aimed to find a valid route the sink. From Figure 5b, we can see that *S* receives the probes sent by *Y*, right after T-III: 404 s and interacts with *Y*, thereby establishing a valid one-hop path from *Y* to *S*. Meanwhile *A* still does its utmost to broadcast the messages of loop detection. Finally, *A* rendezvouses its potential forwarder, *Y*, which can provide a loop-free path for *A*. At T-III: 405.42 s, the loop is removed and the network keeps loop-free until the end of T-III. The relief of loop is basically thanks to the re-connection between *Y* and *S*. This re-connection sharply contrasts with the case in T-II, where *Y* and *S* cannot connect with each other to build a valid link due to the severe lack of handshaking opportunities.

**Remark** **3.** In CTP, a n-node loop (n≥3) will be relieved if any node in this loop can find a forwarder that has a valid path to the sink, because the time-intensive loop-detecting beacons broadcasted by that in-loop node will be responded in short term by some valid forwarder with a high probability; in other words, proactive and timely beaconing in the vicinity of loops would be helpful to alleviate or even remove loops.

## 5. Design of La-CTP

In the previous section we have deeply analyzed the major drawbacks of CTP in handling loops and the corresponding causes. We will detail the design of La-CTP in this section. Essentially, the wrong determination (or update) of parent node makes a loop; so we propose a new metric for parent updating in order to suppress the formation of loops as much as possible. In addition, then we design a proactive and adaptive beaconing scheme which can effectively unlock loops with the cost lower than CTP. Noticeably, La-CTP retains the framework of CTP, the interfaces provided by CTP, and even the parameters used by CTP. Thus La-CTP is totally feasible for any scenarios that support CTP.

### 5.1. Depressing Loops

In CTP, a node makes a decision on parent update according to the logic OR of three conditions, which is shown in Algorithm 1 and the three conditions are denoted by C1, C2, and C3, respectively. The routing engine component of CTP maintains two parameters: currentEtx and minEtx. Once the task of updating route is triggered, the CTP routing engine will immediately scan its routing table to obtain the latest neighbor information, and then determine a new parent. Parameter currentEtx represents the latest ETX value of the current parent, and parameter minEtx, the minimum ETX value that the current node can turn to so far. The three conditional expressions map three different cases. C1 is designed to create neighboring relationship and find the first desirable parent in the network initialization. C2 demands the routing engine to quickly respond to the congestion occurring at the parent. C3 guarantees that the routing engine could always find a best route, without experiencing too much frequent parent updates. Specifically, the two constants of MAX_METRIC and PARENT_SWITCH_THRESHOLD are empirically set to be 0xffff and 1.5 in CTP, respectively.

**Algorithm 1** Determination of parent update in CTP
*◊* *upon the route updating triggered at node u*  Update the local routing tablecurrentEtx = the node ETX of parent of *u* (parentEtx) + the link ETX of the link between *u* and its parent  minEtx = the minimum node ETX value provided by current neighbors  *v* = the neighbor node that can achieve minEtx  minLinkEtx = the link ETX value of the link between *u* and *v***if** currentEtx == MAX_METRIC   or   /∗ condition C1 ∗/    minEtx < parentEtx + 1.0     or   /∗ condition C2 ∗/    minEtx + PARENT_SWITCH_THRESHOLD < currentEtx     /∗ condition C3 ∗/ **then**parent = *v*  currentEtx = minEtx + minLinkEtx  **end if** 
**return**



In experiments we traced the valuation of these three logic expressions; however, we found that C3 overwhelmingly dominates the overall logic OR result in the if statement of Algorithm 1, regardless of whether energy-harvesting nodes are involved. In particular, almost all the loops in experiments running CTP are exactly resulted by the ineffective parent updates determined by the valuation of C3.

**Figure 6:**
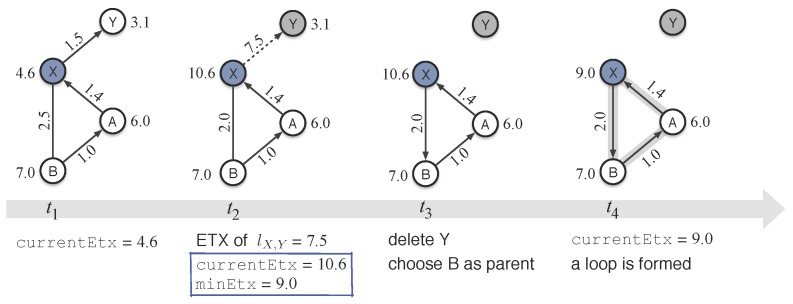
Illustration of a loop caused by conditional expression C3 of CTP which holds true after node *Y* (parent of *X*) is removed out. The numbers near by the node and along the link are the path ETX and the link ETX values, respectively.

Figure 6 shows how the C3 condition of CTP gives rise to a loop in parent updating. At time $t_{1}$, node *X* has three neighbors in its routing table, nodes *Y*, *A*, and *B*; and the parent of *X* is *Y*. At time $t_{2}$, *Y* has left the network and then *X* loses its parent. Continually-failed retransmissions at *X* will increase the ETX value of link $l_{X,Y}$ from 1.5 to 7.5, which will finally trigger the CTP routing engine at *X* to start finding a new route. Before examining the local routing table to determine the neighbor with the minimum path ETX, *X* will first update its routing information. At time $t_{2}$, parameter currentEtx of *X* is changed into 10.6, because *X* thinks that the path ETX provided by its parent, *Y* is still 3.1. By checking the routing entries one by one, *X* can figure out which neighbor is the best and which neighbor should be evicted from its routing table. At time $t_{2}$, *X* believes that *B* will be possibly its best forwarder, which can provide a path ETX value of (2.0 + 7.0) = 9.0; *X* will not choose *A* as its parent because it can easily know that *A* is its previous hop. At time $t_{3}$, the C3 conditional expression is satisfied, so *X* chooses *B* as its new parent—a loop is formed. Even though *X* will update its currentEtx with a smaller value, 9.0, it will surely detect loops once it receives packets sent out by node *A*. Nevertheless, such loops cannot be easily unlocked due to the struggling increases of ETX values at all the three in-loop nodes (see the analyses for Figure 3).

In this paper, we propose a slightly-different but effective condition for deciding the parent updating. The C3 conditional expression of CTP usually fails in choosing another proper parent when the current parent does not work. The essential reason behind is that CTP neglects the following fact. For a node *u* and its parent *v*, the deterioration or the disappearance of link $l_{uv}$ will increase the link ETX value of $l_{uv}$ continually and dramatically, which further increases the current local ETX of *u*, easily making it greater than that of the upstreaming nodes of *u*; therefore, conditional expression C3 will be effortlessly satisfied, followed by the unwise parent updating. To restrain the loop formation as much as possible, La-CTP uses a new condition expression as follows, denoted by C3${}^{\prime }$, to take over the C3 of CTP.

C3^′^: minEtx + PARENT_SWITCH_THRESHOLD < original currentEtx

In the conditional expression C3${}^{\prime }$, La-CTP does not compare the minEtx (provided by the best neighbor) with the latest currentEtx; instead, it compares the minEtx with the original currentEtx, which represents the path ETX value before updating the local routing table. With doing so, C3${}^{\prime }$ cannot easily hold true, even when the current parent is invalid; and then La-CTP cannot easily enter the procedure of updating parent—suppressing the loop formation with descendant nodes involved. It is worth noticing that only the C3${}^{\prime }$ is insufficient to solve the loop problem: it can avoid loop formation as much as possible, but it alone cannot find feasible route for the parent-losing node. In Figure 6, for instance, C3${}^{\prime }$ is false at time $t_{3}$ (C1 and C2 are also false here) and therefore, node *X* will not carry out the parent updating, i.e., *X* will not choose *B* as its new parent. In such a case, however, *X* does not have an idea about how to find another feasible route to deliver its passing-by or locally-created packets. Next we will introduce the beaconing scheme of La-CTP that can recover the routes as long as the network is not segmented permanently.

### 5.2. Handling Loops and Invalid Routes

From the experiment results shown in Figure 4, we can observe that the beacons with too long interval are unable to “awake” the nodes that have been devoured by loops or are joining some loop, although the looping sub-network is not totally separated from the main network topology. CTP employs the Trickle scheme in route beaconing. Specifically, a node will exponentially double its local beacon interval, if it does not experience the parent update, the significant link dynamics, and the request from neighbors for connection. For example, Figure 4b shows that after joining the loop formed by nodes *A*, *B*, and *C*, node *Y* thinks it has already achieved a stable routing and then it doubles its beacon timer. Meanwhile, *S*, the sink node, also slows its beaconing in route maintenance because no traffics arrive by then. Now, *S* and *Y* both broadcast beacons with longer and longer intervals over unreliable channel; the behavior of these two nodes almost devastates the possibility of recovering their re-connection in a short term.

CTP assumes that loops, though unavoidable, are not too many during network; and thus its forwarding engine mainly takes into account the efficiency of network resource, rather than the assisting approaches to resolving loops. The proposed La-CTP uses a more loop-aware timer adjustment scheme in route beaconing, which is described in Algorithm 2. As a well-engineered protocol, CTP synergistically integrates the 4-bit link estimator, the Trickle beaconing scheme, and the ETX-based routing gradient computation. La-CTP follows the structure and the pattern of CTP and only replaces the Trickle scheme of CTP with a proactive and adaptive scheme of adjusting beacon timer. The experiment results show that La-CTP can effectively unlock the loops and recover forwarding paths with low overhead. Algorithm 2 involves three event-driven processes; their critical jobs are to adaptively manipulate parameter $T_{b}$, the period of the beacon timer—when the timer goes off, node will immediately broadcast beacons.

**Algorithm 2** Beacon timer adjustment of La-CTP**Require:**
$T_{b}$: the period of the current beacon timer;             Δt: the interval between the current time and the time of receiving last packet
*◊* *upon the traffic checking timer being fired at u*  **if** Δt≥NO_TRAFFIC **then**    **post** the task adjustBeaconTimer(MIN_INTERVAL)**end if****return** *◊* *upon receiving a packet at u from v*  **if** Δt<NO_TRAFFIC **then**    **post** the task adjustBeaconTimer($T_{b}\times$SCALE_RATIO)**end if****return** *◊* *upon receiving a beacon message at node u from its neighbor v*
Extract this beacon message  **if** *v* is seeking a valid route **then**    **post** the task adjustBeaconTimer(MIN_INTERVAL)  **end if** **if** *v* has already detected loop **then**    **post** the task adjustBeaconTimer($T_{b}/$SCALE_RATIO)  **end if** **return**     **task** adjustBeaconTimer(*t*)      **if** t< MIN_INTERVAL **then**          t= MIN_INTERVAL     **end if**     $T_{b}\leftarrow t+\tau$  /∗τ is chosen randomly from [0, MIN_INTERVAL] ∗/    Stop the current beacon timer and start a new one that will be fired every $T_{b}$ **return**


First, if a node, say *u*, has not received in-going traffics for a longer time, it will shorten its beacon timer to MIN_INTERVAL such that it can proactively catch on to the local network status. By doing so, *u* could save those neighboring nodes already lost in loops as soon as possible, by telling them about “a better way exists”. Different from La-CTP, however, CTP identifies the local network status only by the traffic coming from children nodes, which easily lets the parent node mistaken in thinking “all the upstream network is working smoothly and then I do not need to change anything.” La-CTP uses a threshold, which is denoted by NO_TRAFFIC and set to 2560 ms in implementation. If the node has not received any passing-by packets within Δt and Δt≥NO_TRAFFIC, it will stop its current beacon timer, immediately followed by resetting $T_{b}$ with MIN_INTERVAL and starting a new beacon timer of $T_{b}$.

Second, if a parent node *u* receives one packet from its child node *v* and Δt<NO_TRAFFIC, then *u* will extend its beacon timer SCALE_RATIO times, assuming that the local network runs properly with high probability. Here parameter SCALE_RATIO is a constant empirically set to 2 in the implementation of La-CTP. The exponentially prolonged beacon timer saves the energy of *u* and reduces the possible co-channel conflicts in the vicinity of *u*.

Third, La-CTP shortens its beacon timer, based on the routing messages received. If a parent node *u* receives a beaconing message from child *v*, *u* will first check the current status of *v* encapsulated in this message, and then determine its beacon timer adjustment, depending on the current status of *v*. If *v* broadcasts the beaconing message for seeking new route, *u* will shorten its beacon timer down to the minimum. If the message of *v* tells that it has detected a loop, *u* will shorten its beacon timer by SCALE_RATIO fold. We deal with the new route request and the loop-detection event with different timer adjustment policies because sometimes, the loop-detection event does not always indicate that a loop really happens to the child node. For example, the occasional dramatic link variation in short term affects the link quality evaluation that is made by the ETX component in real time, and then gives rise to a spike of link ETX value, which is possibly followed by a false loop detection. Therefore, La-CTP can wait to see whether loops actually occur at the upstreaming nodes, while shortening its beacon timer step by step. In the case with true loop detection, of course, La-CTP trades off some certain loop-responsing speed for better energy efficiency.

Parameter NO_TRAFFIC lets a node re-connect with its in-loop child nodes in a proactive way, while parameter SCALE_RATIO passively tunes the beacon timer in a packet-driven way, making the node propoerly react to its local network status—continuously prolonging the beacon timer to save energy or shortening the beacon timer to rescue its child nodes from loops. In fact, it is hard to obtain theoretically optimal configurations of NO_TRAFFIC and SCALE_RATIO. Their configurations, open to future developers, depend on the data traffic intensity and the network scale in reality. In experiments, however, we found that the values of NO_TRAFFIC only have insignificant effect on the overall network performance, because inherently, EH-WSNs will not well support traffic-intensive applications. Larger SCALE_RATIO will surely benefit fast loop handling when loops are continually detected at the child nodes, because it can make parent’s beacon timer drop to the minimum more quickly (i.e., less exponential decreases are needed before the minium is hit). However, a larger SCALE_RATIO will make the parent node aggressively react to the false or the sparse loop-detection event by broadcasting periodic messages with the time interval of length MIN_INTERVAL, and then will waste the network resource. In the future we will examine the effect of SCALE_RATIO under certain scenarios and investigate traffic-adaptive and resource-aware update policy of SCALE_RATIO.

It is worth noting that Algorithm 2 uses a *task*, named adjustBeaconTimer, to carry out the beacon timer adjustment. The task is a typical code block in nesC language and the execution of a task can be split from the invoker, thereby be able to enhance the concurrent processing capacity of system. In fact, the syntax of task cannot support any passing-by arguments in nesC, so the argument *t* of adjustBeaconTimer is maintained with a global variable in the implementation of La-CTP. For clarity, however, we specify *t* as an argument of task adjustBeaconTimer in Algorithm 2. Local concurrent beacons will possibly saturate the channel quickly and then lead to communication conflicts easily, especially after loops are formed and reported. To address this issue, we randomize the beacon timer by adding a random offset τ into $T_{b}$. The value τ will be randomly chosen within [0, MIN_INTERVAL] in a uniform manner, whenever the task adjustBeaconTimer is invoked.

## 6. Evaluation

We compare the network performances of CTP and La-CTP with multiple experiments which consist of real wireless sensor motes but realistically simulate the operation of EH-WSNs. We implemented La-CTP based on TinyOS 2.1.2 and nesC codes; so it can support any hardware platforms that can run TinyOS. In this section, we first describe the testbed and evaluation metrics in experiments; second we introduce how the experiments were carried out to simulate the typical EH-WSN operation; finally, we demonstrate and analyze the experimental results.

### 6.1. Experimental Settings and Methodologies

**Figure 7:**
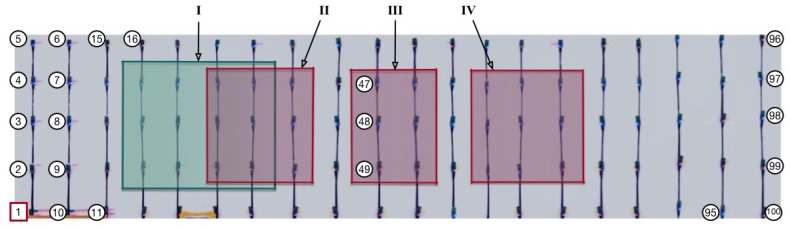
Deployment of the testbed involving 100 TelosB nodes. Node 1 is the sink node that collects the data generated by the other nodes.

Figure 7 shows the node deployment of our testbed, in which 100 nodes are placed in the grid with the interval of about 20 cm and all of them transmit data with a low power (−15 dBm) for the purpose of forming a multihop network. All the nodes are connected, through their on-board USB interfaces, to a USB hub that is powered by the alternating current (AC) of 220 V. By connecting this hub with a PC, furthermore, we can make each node send its runtime information to the PC for storage and subsequent analysis.

Besides of transferring the data from the nodes to the PC, the AC-powered USB hub can supply energy source for all the nodes such that they can keep working or need no recharging at all. To simulate the operation of EH-WSNs, therefore, we pick out part nodes (called the EH-nodes) and let them work with two alternate statuses: the *working status* and the *recharging status*. In the working status, the EH-node can participate into the network operation, including the packet generation, the packet reception, and the packet forwarding. In addition, it can record its runtime debugging logs and send them to the PC. In the recharging status, however, the EH-node does nothing except maintaining an alarm clock; when the alarm clock goes off, this EH-node will switch into the working status immediately. The nodes other than EH-node are called normal nodes, which are always in working status.

We added customized debugging codes in both the source files of CTP and La-CTP. Therefore, we can record every critical operation (debugging information) of these two protocols we were concerned with, and then we can precisely know what happened, though after experiments. Our experiments were carried out under three types of network deployments, which are denoted and explained below. For each scenario, the node numbered 1 was the sink that collected the packets from all the other nodes and transmitted them to the PC for storage.

- noEH: this experiment included 50 nodes with numbers from 1, 2, and up to 50, and all the nodes in use were normal nodes, i.e., without any EH-nodes involved. All noEH nodes were powered by the USB wire.
- EH1: this experiment included 50 nodes with numbers from 1, 2, and up to 50, and only the nodes within region I of Figure 7 were EH-nodes. All EH1 nodes were powered by the USB wire.
- EH2: this experiment included all the 100 nodes and the nodes within regions II, III, and IV of Figure 7 were all EH-nodes. All EH2 nodes were powered by the USB wire.
- AllEH: this experiment included all the 100 nodes and node 1 still served as the sink. Specially, each of the nodes except node 1 was powered by two AA batteries.

In EH1 and EH2, we placed the EH-nodes in the middle part of the network area, in order to form such a network topology: it is close to be physically separate due to the existence of energy-harvesting nodes, while being able to keep connected for those normal nodes. By doing so, we can examine the performance that the routing protocol deals with the loops in relatively extreme cases. In experiments of EH1 and EH2, after the EH-node wakes up, it will stay in working status for 120 s and then enter the recharging status. The communication module and sensors of EH-node will be shut down for a period randomly chosen from 120 s to 150 s, in order to mimic the actual recharging process with uncertain powers. In scenarios noEH, EH1, and EH2, the packet generation rate was set to be 0.1 Hz and each experiment ran 30 min.

Different from scenarios EH1 and EH2, scenario AllEH was designed to simulate an EH-WSN topology that might be transiently partitioned at runtime because of the existence of too many EH-nodes. We conducted the AllEH experiments with following configurations. First, the awake EH-nodes will keep active for 20 s, generating and forwarding packets, and then sleep for a period randomly chosen from 20 s to 30 s. Second, the transmit power of all nodes increases up to −10 dBm, in order to avoid the occurrence of too many segments in the network. In scenario AllEH, each awake node generates packets at the rate of 0.25 Hz. Unlike EH1 and EH2, the debugging logs of each AllEH node were stored in the on-board ROM (read-only memory) which actually is a kind of serial flash chip. Since the Telosb ROM involved in our testbed is only 1024 KB in size, the AllEH experiment only ran 10 min for each protocol, aimed at avoiding the ROM overflow caused by a great amount of runtime log data. After each experiment was done, we transfered the in-ROM logs of each node, through USB wire, to the PC for further analysis. During the initialization of AllEH experiments that had no USB connections with the PC, we let the sink (node 1) broadcast a set of messages with the maximum transmit power (0 dBm), which were used to (1) synchronize the global clock of each node and (2) boot each node with a random delay ranging from 30 s to 60 s such that not all the nodes would enter their first sleeping state at the same time. The time synchronization done only once in the network initialization is sufficient because the local clock skew at each node is reasonably negligible in the experiment that lasted for only 10 min [44,45].

Followed are five metrics used to evaluate the performance of both La-CTP and CTP.

- *Network throughput*: the ratio between the number of packets received by the sink and the number of packets generated during experiment.
- *Number of parent updates*: the total number of parent updates that all the nodes perform during experiment. This metric can reflect the dynamics of the network topology.
- *Number of detected loops*: the total number of loops that occurred during experiment. This metric is used to weigh the performance that the protocol handles the loops.
- *Average time of loop removement*: if parent node *u* determines a loop-detection event just upon receiving a packet from child *v* at time $t_{s}$ and in the following time $t_{e}$, *u* receives another packet from *v*, yet without reporting loop detection, then this loop through *u* and *v* is reckoned to have been removed and the removement time of this loop is calculated by $\left(t_{e}-t_{s}\right)$. We use the average time of loop removements in the routing performance evaluation.
- *Numbers of beacons*: the number of beacons sent by nodes and the number of beacons received by nodes in the routing establishment and maintenance. We use this metric to approximately measure the energy overhead needed by the routing protocol, because the exact energy consumption cannot be measured at the nodes powered by USB lines.

### 6.2. Analyses of Experimental Results

#### 6.2.1. Examination of CTP under noEH and EH1

**Figure 8:**
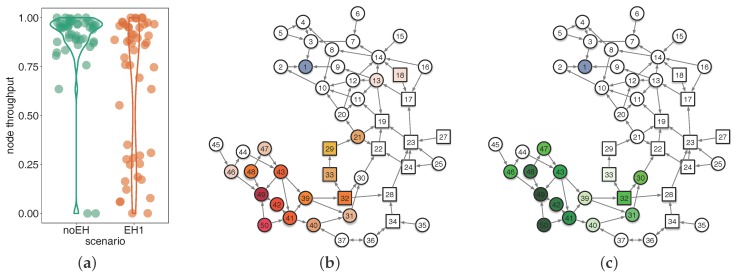
Performance and snapshots of CTP under deployment EH1. In sub-figures (**b**,**c**), square nodes represent EH-nodes (others are normal nodes), node of number one is the sink, the color intensity of a node represent the relative measurements at that node, and the arrows are the directional links being used by CTP when this snapshot is recorded. (**a**) node throughput; (**b**) #parent updates/EH1; (**c**) #loop detections/EH1.

We first conducted two experiments of CTP under deployments noEH and EH1, respectively, in order to examine the effect of EH-nodes on CTP. Then we conducted another two experiments in which La-CTP took over the above two deployments, but we only show part of experimental results in Figure 8 due to the page limit. Figure 8a compares the per-node throughputs of CTP achieved under noEH and EH1. We find that without EH-nodes involved, CTP works very well: most of nodes produce a per-node throughput higher than 86%. When EH-nodes come on, however, CTP deteriorates much in delivering packets to the sink. Even though some nodes can still obtain high per-node throughput, they count for less to guarantee the overall performance because almost half of nodes are lower than 75% in per-node throughput.

Figure 8b,c demonstrate two snapshots taken right before the termination of experiment, which respectively indicate the numbers of the parent updates and the loop detections done at each node. Note here that we visually reshape the network topology in Figure 8b,c, attempting to demonstrate the runtime characteristics of CTP as clearly as possible. In addition, we did not receive any debugging logs from node 38 as expected, and then we did not plot it in the topologies of Figure 8b,c.

We can easily find, from both Figure 8b,c, that if a node has longer paths to the sink and traverses some EH-nodes, such as node 42, 47, or 48, it usually experiences more frequent parent updating and loop detecting. Another observation basically indicates that the further a node is away from the sink, the greater the amount of parent updates and loop detections happened to this node. Actually, those nodes with longer paths to the sink yielded lower per-node throughput due to the existence of EH-nodes on the half way. By the distributions of nodes with higher frequencies of parent updating and loop detecting, we can see an obvious pattern in Figure 8b,c—the nodes undergoing more parent updates usually detected more loops. This is not a simple correlation between parent updating and loop detecting, but a causal relationship, as we have analyzed previously. Here, the experimental results indicate that proper control of parent updates is very necessary to restrain loops in EH-WSNs and is a beneficial start for designing effective routing protocols for EH-WSNs.

#### 6.2.2. Comparison of CTP and La-CTP

**Figure 9:**
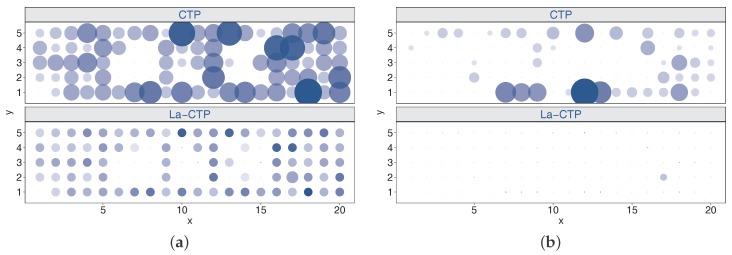
Comparisons in parent updates and loop detections under two protocols running in EH2. (**a**) parent updates; (**b**) loop detections.

**Figure 10:**
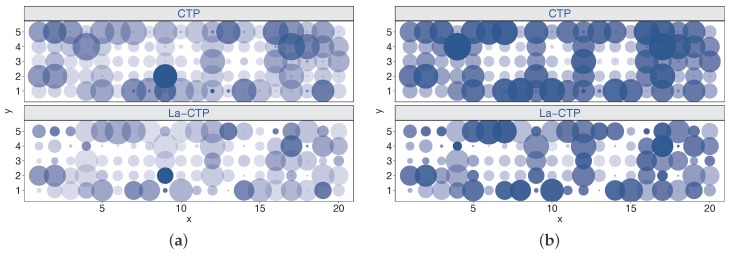
Comparisons in beaconing overhead under two protocols running in EH2. (**a**) beacon sent; (**b**) beacon received.

We conducted experiments with deployments noEH, EH1, EH2, and AllEH to comprehensively investigate the behaviors and the performances of both protocols. Table 1 quantitatively shows the comparison results. From Table 1, we can find that La-CTP is better than CTP in terms of throughput and energy efficiency, even in the scenario without EH-nodes involved. Also, on average, La-CTP needs less time to unlock a loop than CTP. Next we will only give deep analyses of the experimental results achieved by the two protocols under deployment EH2. We use bubble charts shown in Figure 9 and Figure 10 to measure the overheads of the two protocols, where the color and the size of a bubble jointly map to the value it represents. Since per-node measurements achieved by two protocols differ considerably, it is hard to tell how much the differences are, only according to colors and sizes in those bubble charts. We thus omit the legends mapping colors and sizes to values, which leads to little effect on comparing the performance of both protocols.

From Figure 9, we can see that CTP generates more parent updates and loops than La-CTP does. As shown in Table 1, in fact, only 97 parent updates and 11 loop detections happened to La-CTP in the EH2 experiments, both far less than 425 parent updates and 329 loop detections of CTP. Whenever an EH-node enters sleeping status, its previous-hop nodes will update their local variables minEtx and currentEtx and the changes will possibly result in parent updating. When parent updating happens, CTP often mistakenly chooses upstreaming nodes as new parent, thereby creating loops unsurprisingly. In contrast to CTP, the proposed La-CTP evaluates the effect of the network status change, by comparing the currently-updated minEtx with the original currentEtx. Therefore, the La-CTP node will not easily change its expectation or requirement for the forwarder’s capacity, even though its parent cannot provide forwarding services at that moment. La-CTP employs a simple but effective metric for updating parent, so it restrains the opportunity that a node chooses its previous-hop as parent, thus reducing the loops. In return, less loop detections mean that less parent updates will be requested in the subsequent network operation.

According to Algorithm 2, the parent node is required to proactively check the in-traffics coming from its previous-hop nodes, in order to know if they are caught in loops or encounter other conditions. However, CTP only turns to the beacons locally broadcasted, to evaluate the local network status, no matter how long the parent node has not received any passing-by packets due to loops or poor link quality. A speculation comes up naturally: La-CTP employs a more proactive beaconing policy, and does it need more beacon messages than CTP? Figure 10 plots the beacons broadcasted and received in experiments. Different from the speculation made above, La-CTP generates 6756 beacons and receives 92,754 beacons, while CTP generates more than 10,000 beacons and receives more than 138,000 beacons. The reduction of the amount of beacons in La-CTP is mainly attributed to the loop controlling and relieving schemes. Unable to suppress and relieve loops effectively, however, CTP is prone to be trapped in loops and consequently, the nodes detecting loops will always broadcast beacons even without any helpful replies. In some extent, the amount of beacons in network operation can profile the overhead of protocols in terms of energy and bandwidth resources. Therefore, Figure 10 also demonstrates that La-CTP overwhelms CTP in resource efficiency.

Table 1 comprehensively compares the network performances of two protocols under four different scenarios. In the EH2 experiments, when the two protocols terminate, CTP still leaves 54% of loops that were detected during experiments, while La-CTP has already relieved all its loops. In the AllEH experiments, CTP and La-CTP do not solve around 60% and 6% of their loops, respectively. Loops may grab the packets, without forwarding them towards the sink; and finally the packets kept in loops will be dropped finally due to buffer overflow. Hence, CTP cannot achieve a good throughput in EH-WSNs unless it can solve loops properly. We can see from Table 1 that the throughput of La-CTP is almost four times and three times that of CTP in scenarios EH2 and AllEH, respectively. In the AllEH experiments, noticeably, we synthesized the time-stamped neighbor lists of all nodes and found that the network topology was partitioned dozen times at runtime and the largest network segment involved 27 nodes. So, both the parent updates and the loop detections of the two protocols are more frequent in the 10-min AllEH experiment than their counterparts in the 30-min EH2 experiment. In partitioned network, neither CTP nor La-CTP can effectively remove the loops in a segment until one node of this segment could be re-unioned with some nodes that can reach the sink. On average, La-CTP needs 8.8 s to unlock a loop in the AllEH experiment, while CTP needs 20 s to do this.

## 7. Conclusions

The intermittent and random nature of EH-WSN in connectivity motivates us in designing loop-aware routing protocol for real-world EH-WSN applications. Through preliminary simulation experiments, we dissected the structure of the prominent CTP and carefully analyzed its behaviors, and we found that unwise parent updating and unconscious beaconing scheme are the main culprit for CTP to fail in handling loops in EH-WSN. Based on the above observations and analyses, we proposed La-CTP, a loop-aware routing protocol for practical EH-WSNs and conducted extensive testbed-based experiments to evaluate its performance. The experiment results showed that the new parent updating metric of La-CTP can considerably lower the chances of loop occurrence, and that the proactive adaptive beaconing scheme of La-CTP can effectively and efficiently remove the difficult-to-avoid loops. La-CTP can run in a fully distributed way, without any centralized control. Another advantage of La-CTP lies in its strong practicability in engineering because it retains the framework and low-level components of CTP. Some prior works considered the loop issue in their energy efficiency-oriented routings, or focused on designing loop-free routing algorithms with only theoretic performance guarantee. La-CTP is complementary to these works and it can also help fill the gap between the academic evaluation and practical use.

In the future we will evaluate La-CTP in more tangible scenarios that have power-different or heterogeneous environmental energy sources. In addition, we will attempt to employ the transmit power adjustment policy to unlock the loops in the EH-WSNs that are prone to partitioned network topology.

## Figures and Tables

**Table 1:** Comprehensive performance comparison of two protocols under three scenarios.

Metrics	noEH		EH1		EH2		AllEH	
	CTP	La-CTP	CTP	La-CTP	CTP	La-CTP	CTP	La-CTP
Throughput (%)	93.7	94.2	77	91.1	23	84	21.4	68
#Parent Updates	85	86	152	83	425	97	174	39
#Loops Detected	58	15	223	8	329	11	283	56
Loops Unsolved (%)	0	0	36.7	0	54.1	0	59.4	5.8
Avg. Time of Loop Remv. (ms)	1102	788	4217	1792	6035	2811	20,486	8824
#Beacons Sent	762	449	4233	2814	10,972	6756	7752	5069
#Beacons Recv	14,566	10,980	61,095	45,332	138,643	92,754	59,040	31,483
#Total Beacons	15,328	11,479	65,328	48,146	149,615	99,510	66,792	40,552
